# Big Data-Driven Cellular Information Detection and Coverage Identification

**DOI:** 10.3390/s19040937

**Published:** 2019-02-22

**Authors:** Hai Wang, Su Xie, Ke Li, M. Omair Ahmad

**Affiliations:** 1 College of Smart City, Beijing Union University, Beijing 100101, China; 161081210208@buu.edu.cn (H.W.); 171081210207@buu.edu.cn (S.X.); 2 Department of Electrical and Computer Engineering, Concordia University, Montreal, QC H3G IM8, Canada; omair@ece.concordia.ca

**Keywords:** base station almanac, data mining, mobile crowdsensing, network measurement

## Abstract

As one of the core data assets of telecom operators, base station almanac (BSA) plays an important role in the operation and maintenance of mobile networks. It is also an important source of data for the location-based service (LBS) providers. However, it is always less timely updated, nor it is accurate enough. Besides, it is not open to third parties. Conventional methods detect only the location of the base station (BS) which cannot satisfy the needs of network optimization and maintenance. Because of these drawbacks, in this paper, a big-data driven method of BSA information detection and cellular coverage identification is proposed. With the help of network-related data crowd sensed from the massive number of smartphone users in the live network, the algorithm can estimate more parameters of BSA with higher accuracy than conventional methods. The coverage capability of each cell was also identified in a granularity of small geographical grids. Computational results validate the proposed algorithm with higher performance and detection ability over the existing ones. The new method can be expected to improve the scope, accuracy, and timeliness of BSA, serving for wireless network optimization and maintenance as well as LBS service.

## 1. Introduction

For mobile networks, cell (also known as a sector) is the basic unit for partitioning the network coverage area. Base station almanac (BSA) is the core dataset of telecom operators necessary for network operation and maintenance. It describes the basic parameters for all the cells of a network such as the type of a base station (BS), its latitude and longitude, the azimuth and down tilt of each sector inside a BS, etc. As an important data asset and strategic resource of operators, BSA is often of highly confidential, and it is difficult to be acquired by a third party. On the other hand, with the continuous construction, expansion, and optimization of the network, new cells are being deployed and the old ones are removed or relocated. In routine network optimization, the azimuth and down tilt of the sector antennas are very often adjusted to improve the coverage. Therefore, the operators need to maintain and update its BSA database timely.

In general, the network operation and maintenance department of local branches of telecom operators are responsible for the maintenance of BSA and report to their headquarters. Inevitably, there are mistakes and delays during the transmission of the BSA within the organization. Since the BSA information is generally measured manually sit-by-site, it consumes tremendous labor and financial cost. Furthermore, the operators also need to know the BS deployment and the real geographic coverage capability of their competitors’ network. Therefore, how to acquire the BSA database in a timely, accurate, and economical manner is an important issue for the operators.

The third parties other than the operators also need to utilize base station information for the analysis of mobile user behavior, user positioning and other location-based services (LBS). Companies like Apple and Qualcomm utilize end-users’ smartphones to collect the base station information of the mobile and WiFi network, including latitude and longitude of the base station, to provide users with network-aided positioning service [1,2,3]. For example, applications (APPs) can get the latitude and longitude of the cell by calling open an application programming interface (API) (such as www.haoservice.com and www.opencellid.org). However, due to the different purpose of the above methods, the information collected is limited, so it cannot fully meet the needs of mobile network operation.

With the rapid development of smartphone operating systems such as Android and iOS, smartphones have gained worldwide popularity in recent years. According to the 36th China Internet Network Information Center (CNNIC) statistical report on the Internet development in China [4], as of December 2016, the number of Internet users in China reached 731 million, of which 95.1% users surf the Internet with smartphones, far exceeding the usage of traditional desktop computers and laptops (60.1% and 36.8%, respectively). In addition, the rapid growth in the terminal hardware performance including high-speed multi-core processors and a large number of sensors, enables the introduction of many new applications on the phone. Of which, the crowdsourcing-based measurement and applications have received academic and industrial attention. In Reference [5], this kind of new sensing paradigm is named mobile crowd sensing (MCS). Perceptional data collected based on crowdsourcing can be applied in a variety of applications, including mobile network operation [6,7], urban traffic management and monitoring [8,9], environmental monitoring [10], and daily life [11,12], etc. The fast development of MCS provides a new way of network information acquisition and utilization.

In view of the above situation, this paper proposes a mobile network cell information detection and coverage identification method based on network measurement data crowd sensed from end-users’ smartphones. Based on the information acquired by means of monitoring and scanning on massive users’ terminal, the key parameters of the cell in the mobile network are estimated and the BSA database is constructed through the method of machine learning. We evaluate our approach using a large-scale data set collected by MCS system and compare it with some baselines and the state-of-art models. Computational results validate the proposed algorithm with higher performance and detection ability over the existing ones.

The constructed BSA database can be widely used in routine mobile network operation, competitor analysis such as network deployment and coverage capability, and LBS services. The accurately identified quality of the actual coverage provides the operators with a highly efficient way to know the real status, especially the weak coverage area of its network and thus guide the engineers to take actions for improvement. To summarize, the contributions of this paper are as follows:

(1) It escalates the intelligence of network operation for the operators. The proposed method provides with them an effective and efficient way of BSA database maintenance and competitor analysis in two aspects: (a) it improves the accuracy and adaptability of cell location estimation; (b) the more BSA parameters, i.e., the type of BS, sector azimuth, the more cell coverage can be estimated with promising accuracy;

(2) It improves the openness of the network information to the public. The proposed method enables the third parties other than the owner of the network (including other operators and LBS service providers) to acquire the BSA information more freely.

The rest of the paper is organized as follows: Section 2 introduces the related works, including the mobile crowdsensing, base station almanac, the state-of-the-art methodologies of BSA information detection, the MCS-based data acquisition system, and the dataset employed in this paper. Section 3 proposes an MCS-based algorithm for the detection of BSA key parameters. In Section 4, the effectiveness of the proposed method is verified based on the actual data collected from the live network and we compare it with the traditional methods. Finally, Section 5, summarizes the work of this paper and makes some concluding remarks.

## 2. Related Work

### 2.1. Energy-Efficient Mobile Crowdsensing

Reference [5] has made a comprehensive analysis on the collection and application of terminal-side data. In that paper, mobile crowdsensing is divided into two categories: participatory sensing and opportunistic sensing. The former requires active participation of individuals to contribute sensory data (such as uploading photos or reporting the real-time road traffic conditions, etc.), whereas opportunity perception in the form of passive, autonomy work, usually do not require users to actively participate in.

**Figure 1:**
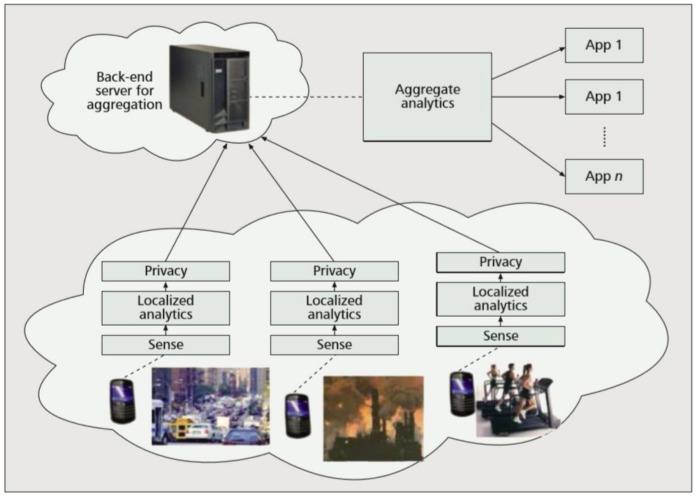
Typical functioning of mobile crowd sensing (MCS) applications.

Figure 1 presents the general framework of an MCS application [5]. Raw sensor data are collected on devices and processed by local analytic algorithms to produce consumable data for applications. The data may then be modified to preserve privacy and is sent to the backend for aggregation and mining.

The challenges of an MCS system mainly lie in two aspects: energy efficiency and effective incentive policy. As we know, that energy efficiency is always one of the key issues in wireless system design [13,14]. Since data sensing and reporting in the end devices always consumes additional energy, how to minimize the energy consumption is a key issue to stimulate the users to participate. Another issue is to design a valid incentive policy to balance the cost of running an MCS system and the data volume contributed by the participants.

In Reference [15], a distributed framework has been proposed for gathering information in cloud-based mobile crowd sensing systems with opportunistic reporting. The data collector periodically broadcasts information about the most urgently needed samples, defined as the data collection utility. Then all participants can determine whether they can contribute the requested data taking into account their cost of sensing and reporting and on the basis of the environmental context. The framework specifies two different data collection policies, a collector-friendly policy and a smartphone-friendly policy, to unify and resolve contradictions between the cloud collector and system participants. Analytical and simulation results validate the energy efficiency of the proposed framework.

Reference [16] has proposed a so-called Piggyback CrowdSensing (PCS) system for collecting mobile sensor data from smartphones that lowers the energy overhead by exploiting smartphone APP opportunities, those times when smartphone users place phone calls or use APPs. In these situations, the energy needed to sense is lowered because the phone needs no longer be woken from an idle sleep state. Similar savings are also possible when the phone either performs local sensor computation or uploads data to the cloud. To efficiently use these sporadic opportunities, PCS builds a lightweight, user-specific prediction model of smartphone APP usage. Piggyback CrowdSensing uses this model to drive a decision engine that lets the smartphone locally decide which opportunities to exploit based on expected energy and quality trade-offs. It is proved that PCS can effectively collect large-scale mobile sensor datasets from users while using less energy (up to 90% depending on the scenario) compared to a representative collection of existing approaches.

Reference [17] presented a survey on the diverse strategies that are proposed in the literature to provide incentives for stimulating users to participate in mobile crowd sensing applications. The incentives are divided into three categories: entertainment, service, and money. Further challenges and promising future directions concerning incentive mechanism design are also discussed in the paper.

### 2.2. Construction of Base Station Almanac

The BSA is the core dataset of telecom operators, which is crucial to the network operation. It describes the basic parameters of all the base stations and cells in a network. Generally, it includes base station name, cell name, base station latitude and longitude, cell identity, type of base station, azimuth of sector, downtilt of sector, base station height, coverage scenario and so on. Among them, the cell identity is a group of parameters, which are different for different network standards. For example, a cell in the global system for mobile communications (GSM), wideband code division multiple access (WCDMA) or time division–synchronous code division multiple access (TD-SCDMA) network is uniquely identified by two parameters, i.e., location area code (LAC) and cell identity (cellID). On the other hand, for long term evolution (LTE) networks, a cell is uniquely identified by three parameters, that is, the tracking area code (TAC), the base station identity (eNodeBID), and the cellID. The type of base station refers to whether the base station to which the cell belongs to is an omnidirectional station (usually only one cell) or a sectorized station (usually, one base station includes multiple cells, also called sectors). Coverage scenario refers to the types of scenes covered by the base station, such as schools, residential areas, commercial areas, rural areas and so on.

Generally, the BSA information is manually collected and reported by the network operation and maintenance department of the local branches of telecom operator. The disadvantages of this approach are obvious:

(1) Erroneous data: Usually it requires a lot of manual participation, which may result in many errors during the process.

(2) Delay: Since the data are reported through many managerial levels up to headquarters, there is always a big delay between the two ends. In this case, the data in the BSA table cannot be fully consistent with the actual situation in the network.

As we know, that BSA is mainly employed by telecom operators for routine network maintenance. With the popularization of smartphones and in view of the various types of location-based services (LBS) being increasingly accepted by the users, BSA is of great value to such applications. It is crucial to network-aided positioning for various LBS services [3], since it can provide precise location information of the base stations. However, considering that BSA is a highly confidential and core data asset belonging to the operator, it is not open to the public. Therefore, the Internet service providers attempt to acquire the key information in BSA by means of network parameter scanning with the terminals. Many methods have been proposed to identify the BSA information, especially the location of BS.

In the early stage of digital mobile communications, a common way to estimate cell tower positions is through wardriving [18]. In wardriving, a vehicle drives within the target area, recording signals radiated from nearby cell towers (or WiFi access points) and the locations these signals were received at. Using this dataset, one can estimate the locations of cell towers with algorithms such as weighted centroid [18] or strongest received signal strength (strongest RSS) [19].

Strongest RSS assumes that the measurement with the strongest observed RSS within a cell, also is the measurement closest to the cell tower. The RSS can potentially be affected by many factors, such as obstacles, other types of radio waves, or the signal receiver in the mobile device. Therefore, this method is simple, yet inaccurate.

Weighted centroid is a simple and effective algorithm for estimating cell tower locations for omnidirectional cells. It estimates the cell tower location to be the geometric center of the measurements belonging to the cell. However, for sectorized cells with a 120° cell span, the correct location of the cell tower is not the geometric center of the measurements.

On top of the strongest RSS and weighted centroid, Yang et al. [20] proposed a cell combining optimization method (CCO). It mainly consists of two steps: RSS thresholding and tower-based regrouping. RSS Thresholding is to filter out all cells whose strongest RSS is lower than a certain cutoff threshold (−60 dBm in this paper). This is to eliminate as many outside cells (i.e., cells fall outside of wardriving area) as possible, since the location estimation of these cells is too poor. Tower-based regrouping technique merges the wardriving traces of cells that share a common cell tower into a single trace and estimating the position of the cell tower itself can improve localization results significantly. It combines the sectors with non-homogeneous coverage into a BS with homogeneous coverage. After applying the cell combining optimization, both the strongest RSS and weighted centroid are improved significantly. Weighted centroid benefits more from the optimization.

With the popularity of smart phones, new cell tower positioning methods have been proposed by employing crowdsourcing data.

Apple Inc. has proposed a positioning algorithm for wireless communication network access point (AP) devices and mobile devices based on the location information of the mobile devices [1]. In this method, the server may receive location information from the mobile devices (e.g., a device with GPS module) of a known location located within the coverage of the wireless AP. Therefore, the position of AP device can be estimated by calculating the geographical average using the received position information of the mobile devices, and when a mobile device is connected to an access point, its location can then be determined based on the position information of the serving AP and the neighboring APs.

**Figure 2:**
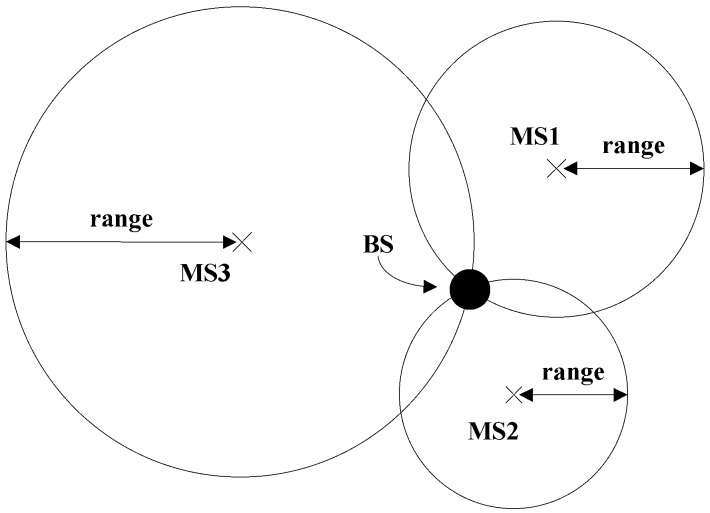
Estimate the location of the base station using the coverage and GPS information of the nearby terminals.

Qualcomm has proposed a weighted centroid method of utilizing the crowdsourced information to improve the base station location information in the BSA database [3]. As shown in Figure 2, the base station estimates the approximate distance (i.e., range in the figure) between itself and the terminals MS1~MS3 by using the collected coverage information (including RSS, Time of Arrival (TOA), etc.) of the serving cell. Taking the position of MS1~MS3 as the center, and the approximate distance between BS and MS1~MS3 as radius, we can draw three circles. Then, the position of the BS can be defined roughly as the intersection position of the circles. Alternatively, the terminal reports its current GPS position if available. The BS aggregates the accurate GPS position of the terminals belonging to the same BS, i.e., taking its clustering center (geometric center) as the BS position and updating in the BSA.

In summary, the abovementioned methods have the following limitations:

(1) Limited application scenarios: As these methods are proposed mainly for the positioning purpose, it detects and updates only those location-relevant parameters of the almanac, that is, the cell ID and the corresponding latitude and longitude of the base station site. It does not tackle other key attributes such as the type of base station, sector azimuth and downtilt of sectors, etc., which are crucial to mobile network operation.

(2) The performance of the Qualcomm method depends heavily on the availability of the coverage parameters of the base station and the GPS positioning of the terminal. It is usually difficult for commercial handset to obtain the coverage information of the neighboring cells and TOA of the serving cell due to its limited access right to low-level database of the handset. In addition, as the signal strength always fluctuates significantly due to the variation of the propagation environment, estimating the position using only these parameters can bring about very large errors.

In addition, although the positioning accuracy of GPS is very high, the actual amount of data available is too small to be effectively utilized. Due to issues like power saving, generally the GPS module is turned on only in case the navigation App starts. Instead, large number of positioning demands are fulfilled through network-aided positioning. Although the accuracy is lower than that of the GPS positioning, the available samples are very rich and can still meet the needs of many application scenarios through a suitable learning algorithm.

**Figure 3:**
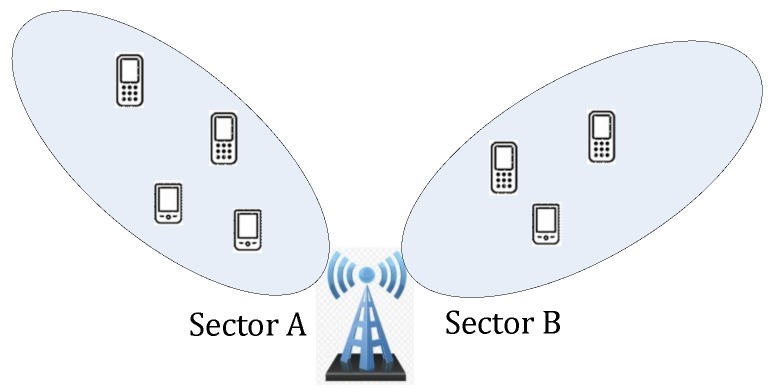
Sectorized BS with two rotational asymmetric sectors.

(3) The weighted centroid methods apply for the omni-directional stations only. It does not deal with the sectorized base station cases, whereas almost all the outdoor stations in urban and suburban areas are sectorized ones. In this case, the non-homogeneity of measurements around the sectorized cell has a big adverse effect. That is, the centroid of the samples does not indicate the cell location.

(4) Although the CCO method is applicable for the sectorized cells, by eliminating the non-homogeneity raised by sectorized cells, it requires the distribution of samples among the sectors of one BS to be balanced, and the sectors of a BS shall be rotational symmetric. When the number of samples in each sector of the same BS varies greatly, the clustering center would shift to a sector with a much larger number of samples, and it possibly results in a larger positioning error. For BS with rational asymmetric sectors, as illustrated in Figure 3 for example, positioning error occurs.

### 2.3. Data Acquisition with Mobile Crowdsensing

Aiming at the shortcomings of the above methods, this paper makes further efforts on the detection and optimization of BSA and proposes a new method. The proposed method is based on the data obtained through a crowdsourcing-based user perception (CUP) system. It is commercially deployed by cooperating with the operator.

**Figure 4:**
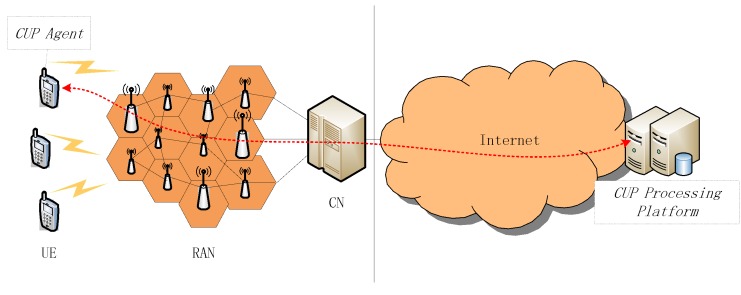
Architecture of the crowdsourcing-based user perception system.

A client–server architecture is employed for the CUP system, as illustrated in Figure 4 [7].

The system mainly consists of two parts: the data acquisition agent in the terminal and the data processing platform at the cloud side. The data acquisition agent, either as a stand-alone APP or a software development kit (SDK) plug-in bundled with other APPs, is deployed in massive number of commercial terminals. Under certain conditions, the agent is then triggered to collect the terminal’s instantaneous wireless parameters periodically by visiting the APIs of the operating system. The sampling period can be configured from the server side. Similar to the energy-saving strategy of PCS [16], the agent is triggered only in case the screen is waked up and user launches the APPs that the agent SDK is bundled with. And a maximum number of samples in one day is configured by the server, so as to further save the energy consumption. The information is transferred to the data processing platform on the cloud side under the pre-defined triggering conditions (e.g., when the terminal is connected to WiFi) through radio access network (RAN), core network (CN), and the Internet, as indicated in Figure 4 with red dotted line.

The sampling dataset of an LTE network includes mainly the followings: date, time, network mode, ID of the serving cell, the positioning information of the terminal, signal strength (RSRP), and signal quality (RSRQ).

The positioning information includes current location (longitude and latitude), the positioning method and precision. In case the GPS module is enabled by the user, the GPS longitude and latitude are collected by the agent. Otherwise, the agent will acquire current location with the third-party network augmented positioning APIs like Baidu or Google positioning. The network augmented positioning is less accurate (normally with errors of several tens of meters in urban area) and consumes less battery than GPS positioning. The agent does not trigger the GPS positioning initiatively, to avoid interference to users and save battery energy.

It shall be noted that, highly sensitive information, such as user ID, phone number, and contents of APPs are not acquired. Thus, there is no violation to user’s privacy. The raw data arrived at the data processing platform are firstly pre-processed, mainly to eliminate invalid data.

One big advantage of the MCS data is spatial homogeneity. As we know, that the samples of wardriving concentrate only on the main roads around the cell. The non-homogeneous spatial distribution of samples always leads to serious deviation of cell location estimation from the actual location. In our data acquisition scenario; however, since the data are acquired from massive number of users throughout the network, the distribution of samples are more homogeneous.

## 3. MCS-Based BSA Information Detection

In view of the limitations of the above-mentioned methods, in this section, we propose an improved BSA information detection algorithm based on the crowdsensing data. It aims at better fulfilling the needs of both LBS services and the network operations. The main target of the proposed algorithm is to estimate the BS type (sectorial or omni-directional BS), cell site location, azimuth of each sector, range of coverage of each cell, and to identify the geographical coverage capability of BS.

### 3.1. Estimation of the Type of BS and BS Location

Samples belonging to the same BS are put together. The type of the base station and its location are then determined based on the geographical distribution and signal strength of the samples in the same BS. Generally, the stronger the signal strength, the closer it is to the base station. A modified weighted centroid method is then employed. Different from the cell combination optimization in Reference [20], a two-stage clustering is utilized to mitigate the influence of imbalance samples among the co-site sectors.

Firstly, all the samples with the same eNBID in the MCS dataset, $D=\{d_{i}|1\leq i\leq S\}$, are grouped together, and denoted as $D_{eID}=\{d_{i}|1\leq i\leq S_{eID}\}\subset D$, where S refers to the total number of samples of the MCS dataset, and *S_eID_* is the number of samples of base station eID. The attribute space of the samples is d = {date, time, TAC, eNBID, cellID, *x*, *y*, RSRP, RSRQ}, whose elements correspond to the date of acquisition, time, tracking area code, base station ID, cell ID, longitude, latitude, signal strength, and signal quality, respectively. Attributes (TAC, eNBID, cellID) are nominal data, while the others are numerical data.

To mitigate the impact of abnormal samples (such as over-coverage samples, and isolated samples drifted from its actual location due to positioning mistakes, etc.) they are removed before the estimation.

By calculating the total distance, which is the sum of the Euclidean distance of each point to all the other points, the average total distance W of the i-th sample can then be obtained as

(1) $$W_{i}=\frac{1}{S_{eID}-1}\sum_{i,j=1,j\neq i}^{S_{eID}}dis({\left(x,y\right)}_{d_{i}},{\left(x,y\right)}_{d_{j}})$$

where *dis*(·) calculates the distance between the two points. We employ the method of Google map to compute this distance:

(2) $$dis((x_{1},y_{1}),(x_{2},y_{2}))=2R\sin^{-1}\sqrt{\sin^{2}(\frac{x_{1}-x_{2}}{2})+\cos x_{1}\cos x_{2}\sin^{2}(\frac{y_{1}-y_{2}}{2})}$$

If the average total distance is greater than a pre-specified threshold Tw, the sample is an abnormal one and is removed from the dataset.

Taking the top *n*% samples of BS that have the largest signal strength, the geometric center $\left(\hat{x},\hat{y}\right)$ is then calculated with their longitude and latitude$\left\{(x_{i},y_{i}),\;i=1,2,\cdot \cdot \cdot ,N\right\}$:

(3) $$\hat{x}=\frac{1}{N}\sum_{i=1}^{N}x_{i},\;\hat{y}=\frac{1}{N}\sum_{i=1}^{N}y_{i}$$

Normally a BS with only one cell is an omni-directional one. In the case of more than one cell within a BS, the type of BS (*tob*) might be sectorized. However, for an LTE network it could also be an omni-directional BS with more than one carrier frequency. In this case, one should make a judgement according to the spatial distribution properties of the samples. Specifically, we can randomly choose *m*% of the samples from the cell in the same BS and count the number of samples *k_i_* (*i* = 1, …, 4) falling in the *i*th quadrant with the geometric center at the origin. Define the statistical deviation δ as

(4) $$\delta =\frac{1}{K}\sum_{i=1}^{4}\left|k_{i}-\frac{K}{4}\right|$$

where K is the total number of samples chosen from the cell. If it is greater than a predefined threshold *T_tob_*, the cell is possibly a sectorized one. If sectorized cells are dominant in a multi-cell BS, then it is a sectorized BS, otherwise an omni-directional BS.

For omni-directional BS, we take its geometric center $\left(\hat{x},\hat{y}\right)$ as the final estimation of the BS location. For a sectorized BS, however, there might be a large deviation between the geometric center and its real location. On one hand, the number of samples in each sector are quite different. On the other hand, there are some special sectorized BS whose coverage are not rational symmetric. For instance, the sectorized BS along a railway is nearly spindle-shaped coverage.

Therefore, the location of a sectorized BS can be estimated by two-stage clustering. First, the initial BS location (*x*_0_, *y*_0_) of each co-site sector is estimated by using Equation (3), and then the final location can be achieved by finding $\left(\tilde{x},\;\tilde{y}\right)$, the geometric center of the initial BS locations of the co-site sectors.

### 3.2. Estimation of Sector Azimuth

For a sectorized BS, an important parameter is the azimuth of each sector. Unlike the BS location, which is nearly stable, it is frequently adjusted during daily network optimization in order to improve the coverage. Thus, it needs to be estimated and updated to the BSA periodically. The estimation of azimuth relies on the chordwise distribution property of all the samples in a BS. Obviously, the key point is to find the optimum boundary of every two neighboring sectors in one BS. Here a chordwise rasterizing method is proposed to find the boundary and then the azimuth of each co-site sectors. It relies on the assumption that, at the boundary of neighboring sectors, the samples may belong to different cells and there is no dominant sector who owns the majority of samples.

**Figure 5:**
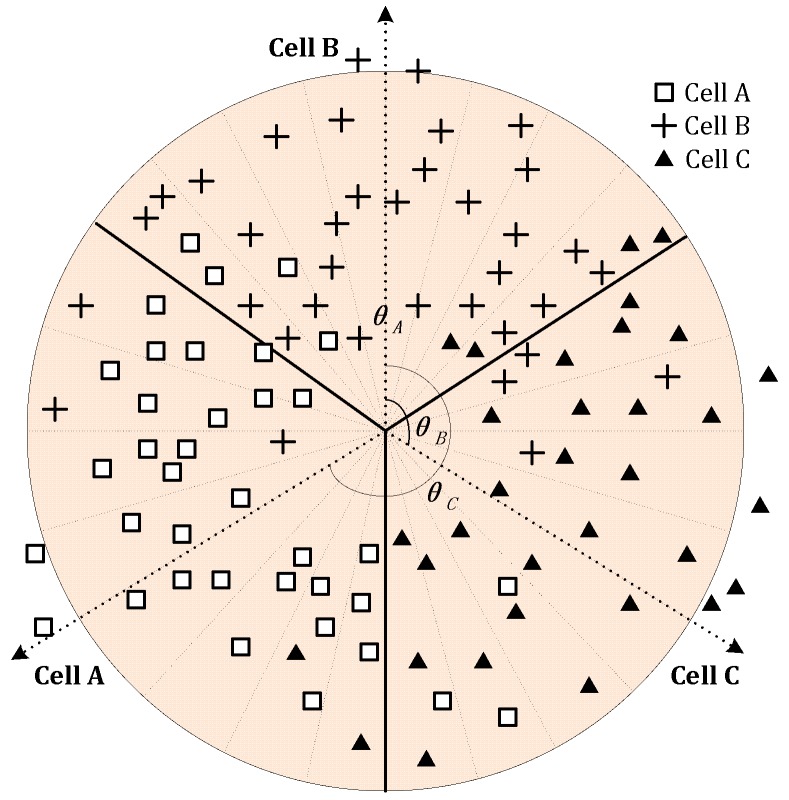
Estimation of azimuth with chordwise rasterizing.

Let us take a tri-sector BS as an example (i.e., a BS with Cell A, B, and C), as illustrated in Figure 5. Firstly, the BS coverage area is chordwise divided into radial rasters with equal raster angle, say 15°.

Select a certain portion of samples in the BS, equally from each sector. If the number of samples of one cell in the radial raster exceeds *Tc* of the total number of samples in the area, the raster is judged to belong to this cell. Otherwise, it is judged as a boundary raster.

We then define the north angle of the central direction of all the continuous neighboring rasters belonging to one cell (excluding the boundary rasters) as the azimuth of the cell. For example, in Figure 5, *θ_A_*, *θ_B_*, and *θ_C_* refer to the azimuth of cell A, B, and C, respectively.

### 3.3. Cell Range Estimation and Coverage Identification

As mentioned above, the stronger the signal strength is, the closer it is to the base station. Then, the cell range can be estimated according to the geological distribution and signal strength of the samples in a cell.

Specifically, samples with RSRP exceeding −110 dBm (usually −110 dBm is the weak coverage threshold set for a LTE network), i.e.,$\left\{d_{i}|RSRP\geq -110\right\}$, are taken as effective coverage samples of the serving cell. Taking the top 10% effective coverage samples that are farthest from the base station, the average distance from these samples to the base station is defined as the cell range γ, i.e., the effective maximum coverage distance is given by

(5) $$\gamma =mean\left\{max_{top10\%}dis\left(\left(\tilde{x},\;\tilde{y}\right),\left\{d_{i}|\mathrm{RSRP}\geq -110\;\right\}\right)\right\}$$

Furthermore, identifying the overall coverage capability of all the base stations in the entire coverage area is always a very attractive topic for network maintenance and optimization. Huge amount of crowdsensed data with positioning information make it possible to identify the BS coverage capability precisely.

Firstly, the target area is divided into grids of 50 m × 50 m or 100 m × 100 m. The latitude and longitude of the center for each grid is calculated and recorded. Then, for every single grid whose number of samples is greater than a pre-specified threshold T_s_, (note that there may be grids with very few samples if the cell phones do not enter the grid, or when the signal of the grid is too weak), we can identify the coverage quality and dominant serving cell of each grid by calculating the average signal strength and the coverage probability of all the nearby cells whose samples fall into the grid.

The calculation of coverage probability can be carried out as follows: Firstly, calculate the number of samples and average signal strength of each cell (i.e., S*_i_* and R*_i_*, *i* = 1~M), and the distance between the center of the grid and each cell site (i.e., D*_i_*, *i* = 1~M). Then, the number of weighted samples for each cell can be obtained using

(6) $${\tilde{S}}_{i}=S_{i}\cdot {\mathrm{ru}}_{\left(R_{i}\right)}\cdot {\mathrm{rd}}_{\left(D_{i}\right)},\;i=1\sim M$$

where ru_(R*i*)_ denotes the ranking of R*_i_* within the group arranged in ascending order, and rd_(D*i*)_ denotes the ranking of D*_i_* within the group arranged in descending order.

Define the cell coverage probability as the ratio of the number of weighted samples of a cell to the total number of weighted samples in the grid. If a cell with coverage probability greater than the threshold *T_c_* (default value 50%), it is then marked as the “dominant cell” of the grid. Otherwise, the grid is marked as “non-dominant grid”.

There may exit an isolated grid (i.e., its dominant cell is different from that of the surrounding grids), due to a poor positioning accuracy or insufficiency of the number of samples in the grid. In this case, a post-processing is needed to smooth the judgment, by considering the cell that dominates the maximum number of grids surrounding the isolated grid, as the dominant cell.

### 3.4. MCS-Based BSA Information Detection Algorithm

**Figure 6:**
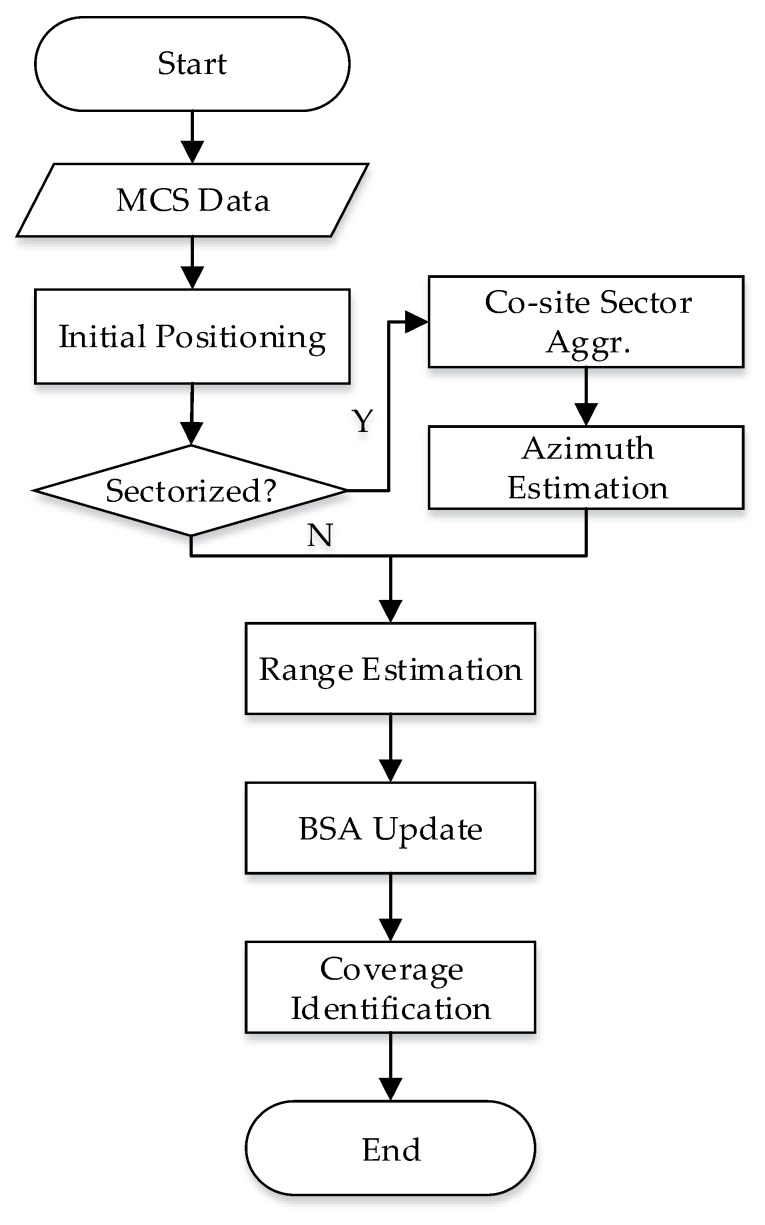
Flowchart of BSA detection algorithm.

The flowchart for the overall process of BSA information detection and coverage identification is given in Figure 6.

The detailed algorithm of BSA information detection is described as follows.


**Algorithm 1. BSA information detection and coverage identification**

**Input:**
$D=\{d_{i}|1\leq i\leq S\}$
1. **for** eNBID ∈ {eNBIDs in D} **do**2.    Remove abnormal samples according to Equations (1) (2);3.    Calculate the geometric center $\left(\hat{x},\hat{y}\right)$ according to Equation (3);4.    Determine *tob* according to Equation (4);5.    **if** (*tob*==sectorized) **then**6.         **for** cellID ∈ {cellIDs of the eNBID} **do**7.         calculate initial BS location (x_0_, y_0_);8.         estimate the azimuth *θ* θaccording to Section 3.2;9.         $(\tilde{x},\tilde{y})\leftarrow$ geometric center of (x_0_, y_0_) for all the co-site cells;10.   **else**
$\left(\tilde{x},\tilde{y})\leftarrow (\hat{x},\hat{y}\right)$;11.   Estimate cell range γ γaccording to Equation (5);12. Divide the coverage area into grids {*G_i_*, *i* = 1~N};13. **for** each grid whose number of samples > *T_s_*
**do**14.   **if** (average signal strength of the grid<−110dBm) **then**15.     mark the grid as “weak coverage grid”;16.   **else**17.     **for** each cell who has samples in the grid **do**18.        calculate S*_i_* and R*_i_*, and Di;19.        calculate ${\tilde{S}}_{i}$ Siaccording to Equation (6);20.        calculate cell coverage probability P*_i_* for each cell;21.        **if** (P*_i_* > T_c_) **then**22.           mark the cell *i* as “dominant cell” of the grid;23.        **else** mark the grid as “non-dominant grid”;24. Smoothing the judgment of dominant cell for each grid;25. Update {eNBID, cellID, *tob*, $\tilde{x}$, $\tilde{y}$, γ , θ} to BSA;26. Output coverage identification results of each grid.

## 4. Experimental Results and Analysis

In order to evaluate the performance of the proposed algorithm, the BSA parameters and coverage are estimated with the proposed algorithm and the crowdsourced samples in the live network and compared with the parameters in the real BSA database.

The data was acquired with the CUP system in the LTE network of Shanghai in 2017. A preprocessing was carried out to deal with errant data including duplicated data, missing values and outliers. The number of effective samples after preprocessing is 109,185,741, covering 9658 base stations. Considering the large size of the data, a Spark cluster consisting of 4 nodes was utilized for the experiment. Each node was equipped with 16 cores and 128 GB RAM.

### 4.1. Criteria for Performance Evaluation

The following indicators were defined to evaluate the performance of BSA estimation, which specifically relate to the accuracy of BS type estimation, the deviation of cell site location, and the antenna azimuth estimation.

#### 4.1.1. Ratio of Correctness of BS Type Estimation

It was defined as the ratio of base stations whose type were judged correctly to all the base stations, that is,

(7) $$R_{B}=\frac{{\tilde{N}}_{B}}{N_{B}}\cdot 100\%$$

where $N_{B}$ and ${\tilde{N}}_{B}$ were the total number of base stations, and the number of base stations whose type was correctly judged, respectively.

#### 4.1.2. Absolute Deviation of BS Location Estimation

The absolute deviation of BS location estimation, $\delta_{A}$, is defined as the Euclidean distance between the estimated and actual BS location (assuming the BS location in BSA is the true location). The Euclidean distance is calculated using Equation (2).

#### 4.1.3. Relative Deviation of BS Location Estimation

Since the designed target coverage of base stations in different coverage scenarios are different, the coverage of base stations in dense urban areas is generally much smaller than that in rural areas. Therefore, the absolute deviation of BS location estimation in different scenarios cannot fully describe the actual performance; therefore, we further define the relative deviation of BS location estimation as

(8) $$\delta_{R}=\frac{\delta_{A}}{\gamma }\cdot 100\%$$

#### 4.1.4. Average Error of Azimuth Estimation, $\bar{\epsilon }$

This index is defined as the average absolute value of the azimuth estimation error (assuming that the azimuth in the BSA is ground-truth) for all the sectors.

### 4.2. Evaluation and Analysis of Results

**Figure 7:**
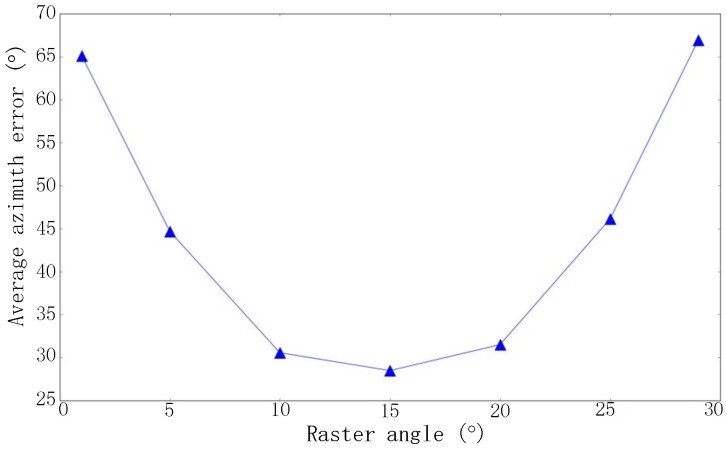
Average estimation error of azimuth in case of different setting of raster angles.

All the thresholds and parameters involved in the proposed algorithm can be determined either based on the statistical analysis of the actual dataset acquired in the live network, or based on the experience. For example, the raster angle in the chordwise rasterizing is determine by experiments. Generally, the smaller the angle is, the more precise the boundary can be judged. In case the samples in a BS is limited, however, too small raster angle will lead to very few samples in a raster and thus wrong judgement of boundary. As seen from Figure 7, 15° gives the optimum results, which is calculated with 387 base stations in the dataset. Thus, we take 15° as the raster angle in the following experiments.

The value of the main parameters in the experiments is given in Table 1.

#### 4.2.1. Performance Analysis of a Single BS

We first randomly select two base stations each with sufficient number of samples, a suburban station A and an urban station B, respectively (both being sectorized), from the data set. The fundamental information of them in BSA is given in Table 2. Accordingly, number of samples in each cell of station A is (4100, 86,100, 5547); and (10,724, 7281, 8556) in each cell of station B.

**Figure 8:**
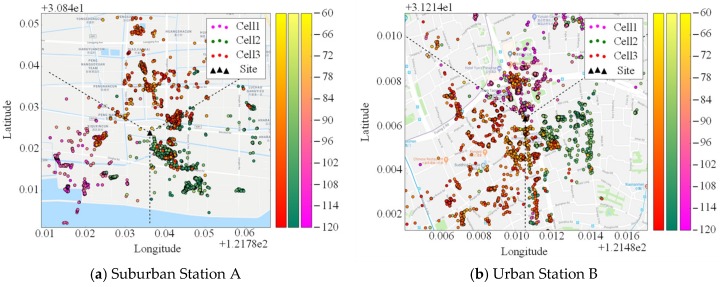
Spatial distribution of the BS and samples (dotted line is the border of co-site sectors, the angle bisector of each sector is the direction of antenna azimuth). (**a**) Suburban Station A; (**b**) Urban Station B.

Figure 8 presents the distribution of the real location and samples of the base stations. Black triangle indicates the location of the BS site. Samples with different color belong to different sectors.

**Figure 9:**
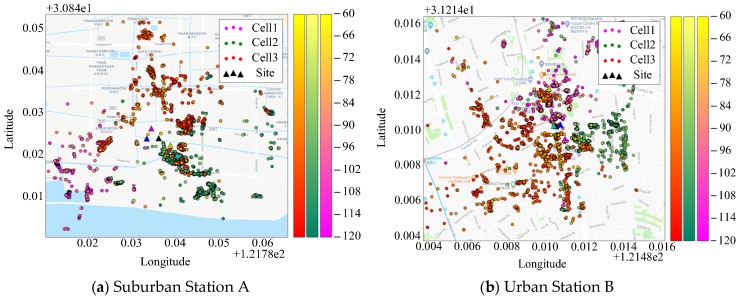
Comparison of BS location estimation.

(a) The locations of BS A and B are first estimated using the proposed Algorithm 1, the CCO method [20] and the Qualcomm algorithm [3], and then, their locations are queried through the API provided on the website www.haoservice.com. The results are shown in Figure 9.

In the figure, the black triangle is the real location of BS according to the BSA, while the blue, yellow, magenta, and cyan triangles indicate the BS locations estimated by the proposed algorithm, haoservice website, CCO, and Qualcomm algorithm, respectively. Detailed results of the algorithms and their absolute and relative deviations from the real BS locations are listed in Table 3.

It can be seen that, in general, the estimation provided by the proposed method is more accurate than those provided by the other methods. However, for the estimation of a suburban station, both the absolute and relative deviations are obviously inferior to the results of urban base stations. The most likely reason for this is the difference in the positioning accuracy of the samples. As mentioned above, most of the collected sample location information comes from network-aided positioning rather than GPS. In this case, the more densely available base stations and WiFi access points are, the higher the accuracy of network-aided positioning is. Therefore, the positioning accuracy of the samples in an urban area is generally higher than that in suburban and rural areas.

**Figure 10:**
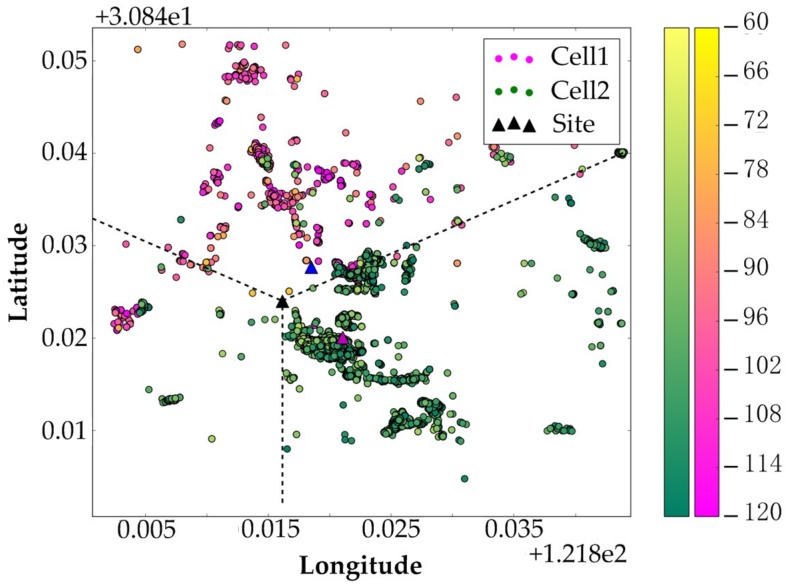
Comparison of BS location estimation for Bi-sector station.

Suppose suburban station A is a bi-sector one similar to that in Figure 3, we estimate its location with CCO and the two-stage clustering method. The estimated locations of the two methods are (121.82107, 30.86003) and (121.81849, 30.86764), corresponding to an absolute deviation of 644 m and 465 m, respectively. The results are illustrated in Figure 10, where the blue and magenta triangle represent the estimated locations of CCO and the proposed method. Although a degradation can be observed for the proposed method compared with that of the tri-sector case, the new method still performs much better than CCO. Therefore, the proposed method is significantly superior to CCO in dealing with radial asymmetric BS.

**Figure 11:**
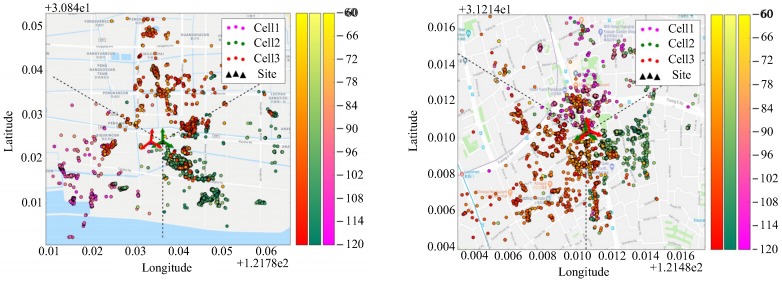
Azimuth estimation results: (**a**) suburban station A, (**b**) Urban station B.

(b) The cell azimuth of the above two stations are estimated using the proposed method and compared with the original data in the BSA. The result is shown in Figure 11. The green arrow in the figure is the real azimuth in the BSA, and the red arrow is the estimated azimuth of each sector. The estimated azimuth of the three sectors at station A are 7°, 120°, 232°, with an average error of 5° compared to the BSA table, and the estimated values of the sectors at station B are 345°, 112°, 232°, with an average error of 10.3 °. It can be seen that the estimation error of the urban area is obviously higher than that of the suburban area. A relatively small volume of the samples may be one of the reasons. More complicated scattering propagation environment of urban area might be another reason.

(c) Impact of sample size on the performance: In the actual data collection process, due to the randomness of terminal distribution and movement, the actual number of samples under different base stations is greatly deviated, and in some cases the number of samples are insufficient. Therefore, in the following, we make a brief comparative analysis on how the volume of samples affects the accuracy of estimation. To simulate the cases of small sample volume, we choose randomly 50,000, 25,000 and 6000 samples from the set of all the samples of the suburban station A and estimate the BSA parameters. The results are given in Table 4.

The error in the azimuth values gives in the table is averaged over all the three sectors in BS. It can be seen that, as the sample size decreases, the estimated deviations of the BS location and the azimuth values increase. In particular, when the number of samples is reduced to 6000 per BS, the performance degradation is particularly noticeable.

#### 4.2.2. Statistical performance evaluation of BSA estimation

According to the above analysis, a good BSA information estimation can be obtained when the sample size of BS exceeds 10,000. Therefore, a total of 4037 stations whose number of samples exceeds 10,000 are calculated using the proposed BSA information detection algorithm. The estimation results averaged over all the base stations are given in Table 5. Among them, the accuracy of BS type estimation is close to 100%, indicating that the algorithm has good performance in finding the type of BS. The estimation of BS location and sector azimuth also provide promising results.

**Figure 12:**
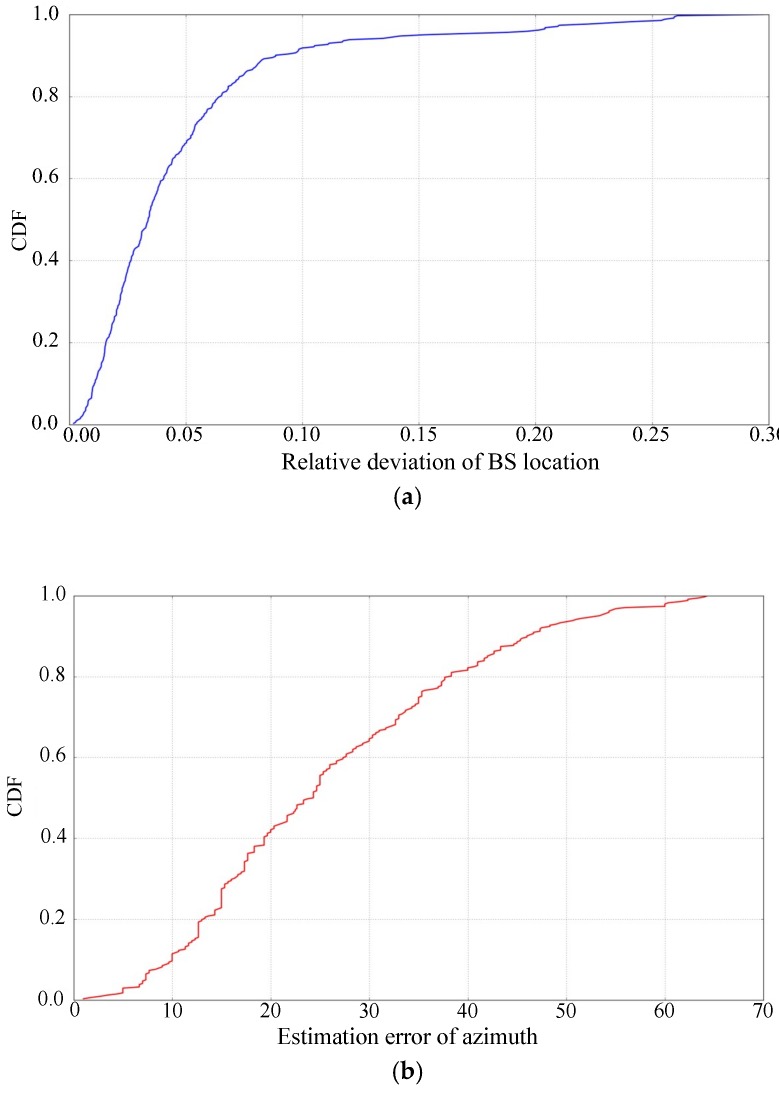
CDF of BSA estimation: (**a**) Relative deviation of BS location; (**b**) Estimation error of azimuth.

In order to observe the performance of the proposed algorithm more closely, we further calculate the cumulative distribution function (CDF) of BS location and azimuth estimation and show them in Figure 12.

It can be seen from this figure that for 80% base stations the relative deviation of BS location is less than 5.3% and the estimation error of azimuth is less than 38°. This shows that the proposed algorithm provides promising results. In some cases, however, there are still big deviations from the actual BSA, and needs to be further optimized.

#### 4.2.3. Analysis of Coverage Identification

**Figure 13:**
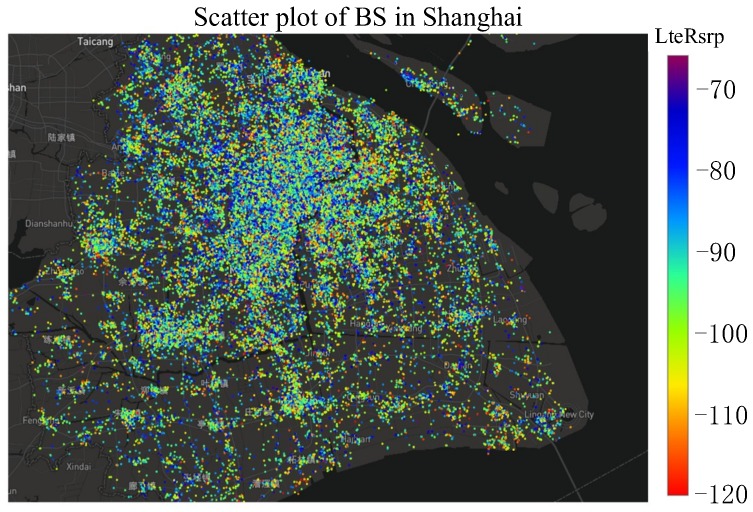
Scatter plot of samples of whole city.

Firstly, to have an overall indication of the quality of coverage in the target area, all the samples are plotted on the map of Shanghai and illustrated in Figure 13. The color of each sample represents its signal strength (i.e., value of RSRP in dBm). A red point represents weak coverage.

Taking a 1000 m by 1000 m square area in the city with totally 97,942 samples as an example, we have the following analysis with the proposed coverage identification method. The area is divided into square grids, where the grid size is carefully selected to reach the best compromise between the statistical significance and local feature. Larger grid size leads to larger average number of samples in each grid. Then a higher statistical significance in the coverage analysis can be achieved at the expense of the loss of local features. On the contrary, very few samples in a grid will inevitably result in statistical insignificance.

**Figure 14:**
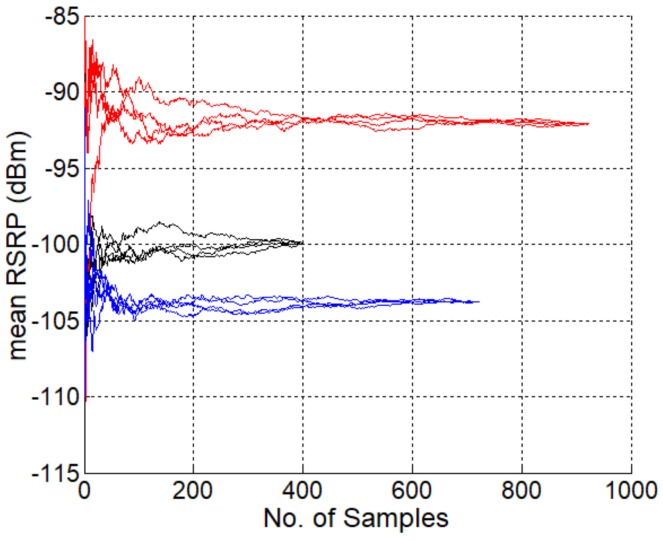
Mean RSRP with the increase of number of samples.

For a 50 m by 50 m grid partitioning, on average we have 244 samples in each grid. Figure 14 presents the mean RSRP of three grids chosen from the area, each with 401, 724, and 924 samples. Four curves are plotted for each grid by shuffling the samples. It can be seen that, with about 200–300 samples, a statistically stable RSRP can be achieved. If a 20 m by 20 m grid partitioning is employed, then each grid has only 39 samples in average. Therefore, a 50 m by 50 m grid partitioning is appropriate in the coverage analysis.

**Figure 15:**
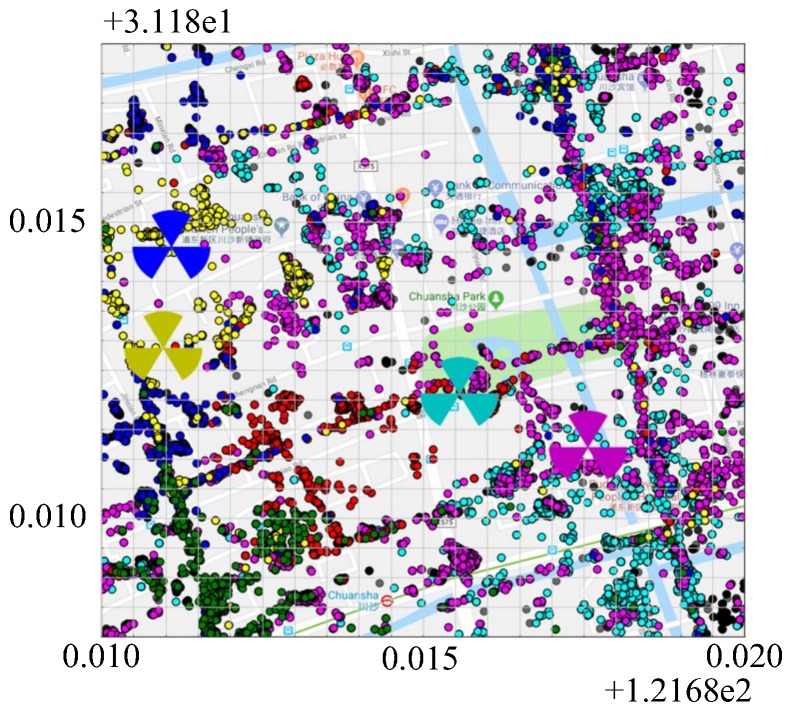
Scatter plot of samples of target area.

Figure 15 shows the distribution of the samples and the location of the relevant base stations in this area. Red, yellow, blue, green, cyan, and magenta points belong to six different BSs, respectively. The black points result from the base stations with very few samples in the target area. The tri-sector icon indicates the BS location whose color is same as the related samples. The direction of each sector refers to the sector azimuth of the BS. As the BS of red and green samples are not within the scope of the target area, the icon is not presented in the figure.

**Figure 16:**
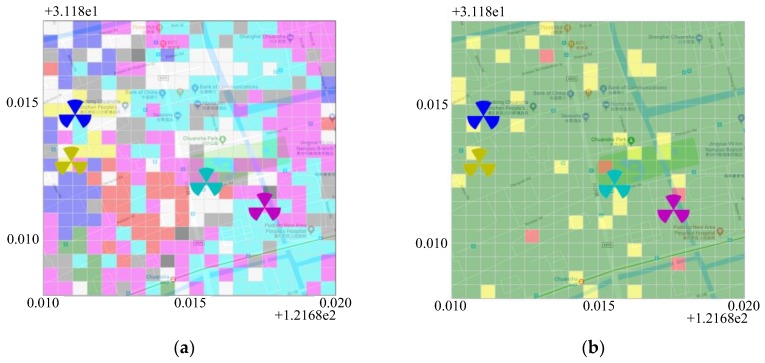
(**a**) Coverage identification of target area, (**b**) Cross-operators comparison of coverage.

Figure 16a presents the coverage identification results of the target area. The white grid indicates there are not enough samples in the grid, i.e., an “unknown grid”. The gray grid indicates that the average signal strength is less than −110 dBm, i.e., a “weak coverage grid”. Black grid refers to a “non-dominant grid” that has no dominant serving cell. Otherwise, the other color of grids indicates the dominant serving BS of the grid.

From Figure 16, we can clearly see that the overall coverage in this area is good. Most of the grids have their dominant serving BS with qualified signal strength. However, there are still a few numbers of weak coverage grids and unknown grids, indicating that more work needs to be undertaken to further improve the coverage of the area. In addition, note that many grids with different colors are intersecting with one another, thus requiring further analysis. Possible reasons might be the overlapping coverage of base stations with different carrier frequencies, or indoor and outdoor base stations.

In the case having samples of different operators’ network in the same area, a cross-network coverage benchmarking can be made. Figure 16b shows the comparison of the coverage quality of the abovementioned LTE network with respect to other two networks of competing operators in the same area. The average signal strength of each grid of every network is calculated. The color of the grid is shown as green, yellow, or red, respectively, indicating that the coverage quality of the target network is the best, medium, and worst among all the three LTE networks. In this example, we can see that most of the grids are green, some yellow and very few are red, showing that the overall coverage quality of the target network is extremely good. This figure provides a simple visualized indication as to which area should be further optimized.

## 5. Conclusions

In this paper, an algorithmic framework utilizing mobile crowdsensed data has been proposed. It enables the engineers to identify the key BSA parameters and coverage capability instead of time-consuming on-site measurements. Concretely, we first develop a probabilistic model that can estimate the type of BS. Then we propose a two-stage clustering method to estimate the location of BS and a chordwise rasterizing method to estimate the sector azimuth. Finally, we calculate the cell range and analyze the coverage capability with a statistical-based method.

Based on the network-related data crowd-sensed from the live network, the performance of the proposed method is evaluated and compared with that of the existing methods.

In the BSA parameter estimation, judgement of the type of BS is relatively simple. It can be made according to the homogeneity of the chordwise distribution of all the samples in a BS. Experiments show that the accuracy can reach 99.9%. While the estimation of BS location and sector azimuth is more important and difficult. Firstly, because the proposed method involves the setting of many parameters, we need to determine the optimum parameters through experiments or experience. For important parameters such as raster angle, experiments are carried out to find the optimum values. The other parameters basically are determined by experience, and there should be room for optimization.

On this basis, experiments of BS location estimation are carried out with two typical urban and suburban BS. Both of them are tri-sector base stations. The results show that the proposed method is obviously superior to the existing methods in estimation accuracy of station location. For omni-directional BS, the method is then degraded to the traditional weighted centroid method. The estimation is more accurate for urban base stations than that it is for suburban ones, which in general have larger cell range.

The proposed method also shows satisfactory estimation ability in determining the sector azimuth. Since there are no other benchmarking methods, we calculate only the deviation between the estimation results and the actual value in the BSA. The deviation of urban station in the experiment is significantly higher than that of suburban station. The fact that urban stations have experienced much complicated multi-path environment and the relatively smaller sample size might be the reasons in the experiment. In the statistical analysis of 4037 base stations, the average deviation is 25.7 degrees, which shows that the method is feasible, but there are still room for optimization so as to satisfy the needs in network optimization.

In addition, considering that the performance of weighted centroid method depends largely on the spatial distribution of samples, the density of samples usually has a great impact on the performance. Therefore, we compare the results of BS location and sector azimuth under different sample sizes. The results validate our expectation. That is, with the increase of samples, the spatial distribution of samples can better represent the actual cell coverage capability, and thus leads to a higher accuracy of estimation. Finally, the reasonable value, i.e., 10,000, of the minimum sample size of a BS in the BSA estimation is obtained.

The coverage analysis in this paper includes the estimation of cell range, and the grid-wise coverage analysis. The latter refers to the identification of weak coverage area, non-dominant grid and cross-network benchmarking. The analysis can provide a valuable guidance for network coverage optimization, including (1) finding weak coverage areas by grouping all the adjacent weak coverage grids; (2) finding unreasonable remote coverage grid: if the distance between a grid and its home BS exceed a certain value or there are other BS in between them, then mobile phones in such grid will experience unnecessary frequent handover; (3) finding non-dominant coverage area: there is no cell in this area which is obviously superior to other cells to provide coverage. All of above will lead to unnecessary frequent handover and escalate the risk of service interruption. It is necessary to adjust the antenna parameters of the neighboring base stations in order to provide coverage for the grid area; (4) finding the competitive disadvantage areas: how to maximize the efficiency of capital expenditure with limited funds in network optimization? An effective way is to find the coverage area which is inferior to its competitors’ and improve it by adjusting the base station parameters or adding new BS. It is an important means for operators to improve or maintain its network capability and competitiveness.

In this paper, we have done some preliminary work in the above aspects, mainly the estimation of cell range and the identification of abnormal grids, which provide a basis for more intelligent coverage analysis. Specifically, first of all we determine the reasonable granularity of gridding by statistical analysis, taking into account the statistical validity and retaining the local features as far as possible. On the basis of gridding, we carry out coverage analysis and grid labelling and cross-network benchmarking for the selected 1km^2^ area. From the visualization results, we can basically evaluate the coverage capability of the target area through the above analysis. It provides a valuable guidance for network optimization work. However, the experimental results have not been validated with the field measurements. More comprehensive and intelligent analysis is necessary to accomplish the above tasks.

In summary, we made a preliminary exploration on how to utilize massive crowdsourcing sensing data to estimate the key parameters of BSA and evaluate the network coverage and proposed a relatively complete algorithmic framework. It will play a positive role in escalating the intelligence of network operation and maintenance. It will also improve the openness of network information for the third party besides the owner of the network, so that the third party can construct an accurate BSA information table through this method for LBS service or competitor analysis.

However, still there are many aspects need to be improved.

(1) The performance of the proposed method depends heavily on the scale of MCS data. While the scale of data depends on how to deploy a large number of CUP agents to user terminals and ensure high activity. Therefore, how to deploy the CUP agents widely and acquire massive data is always a difficult task;

(2) Estimation of the location of indoor BS: because the location of samples utilized in this paper is mainly by network-assisted positioning, only less than 10% of the samples are by GPS positioning. The accuracy of longitude and latitude is relatively low, and there is no altitude information. For indoor BS with only tens of meters coverage, estimation of its location is very much sensitive to the location accuracy of the samples. What is more, the location of BS on different floors also needs to be distinguished. All of this leads to serious estimation error. Therefore, the proposed method is only applicable to outdoor macro base stations. How to locate the indoor BS accurately is still an unsolved challenge. Utilizing the fingerprint of WiFi signal in each floor of the building might be a solution;

(3) The performance of the azimuth estimation algorithm still needs to be optimized: The estimation of sector azimuth depends on the accurate estimation of the boundary of the adjacent sectors in a BS. It is a typical classification problem in machine learning. The average performance of the chordwise rasterizing method in this paper still needs to be improved. More favorable machine learning algorithm are to be employed;

(4) The grid-based coverage analysis needs deeper and more intelligent algorithm. This paper makes a preliminary exploration on the labelling of abnormal grids and requires further manual analysis by engineers to guide network optimization. How to analyze the anomaly network more intelligently and find out the network problems, such as the identification of remote coverage area and the aggregation of weak coverage grids, need to introduce stronger machine learning algorithm.

In the future, we will further optimize the algorithm to improve the estimation accuracy of the key parameters and the coverage identification. In addition to the crowdsourcing data collected by the terminal side, data that may also be utilized include deep packet inspection (DPI) and measurement report (MR) data acquired from the network side. These data have the advantage of much larger data volume, and the disadvantage of missing the positioning information or reduced accuracy. How to combine these data with MCS data to better serve the BSA detection and coverage identification is our focus for future research. Moreover, optimization of algorithm parameters is also required, and may still have a greater impact on performance.

## Figures and Tables

**Table 1:** Settings of Algorithm Parameters.

Parameter	Value	Parameter	Value
$T_{w}$	1.5 W	raster angle	15°
$T_{tob}$	0.4	*n*%	10%
$T_{s}$	50	*m*%	20%
$T_{c}$	50%		

**Table 2:** Fundamental Information of BS A and B in BSA.

BS	TAC	eNodeBID	CellID	Long, Lat	Azimuth
A	23360	374111	(49, 50, 51)	121.81615, 30.86398	(0°, 120°, 240°)
B	23312	382115	(52, 53, 54)	121.49054, 31.22026	(0°, 120°, 240°)

**Table 3:** Estimate Results of BS Location.

	Suburban Station A			Urban Station B		
method	Long/Lat	*δ_A_* (m)	*δ_R_*	Long/Lat	*δ_A_* (m)	*δ_R_*
BSA	121.81615, 30.86398	-	-	121.49054, 31.22026	-	-
Qualcomm	121.82044, 30.85984	617	-	121.49023, 31.22029	21	-
CCO	121.81429, 30.86637	320	-	121.49102, 31.21953	93	-
haoservice	121.81864, 30.86795	502	-	121.49017, 31.22240	241	-
proposed	121.81343, 30.86391	260	7.9%	121.49076, 31.22036	24	1.2%

**Table 4:** Performance Comparison for Different Sample Volumes.

Total Amount of Samples	*δ_A_* (m)	*δ_R_* (%)	$\bar{\in }$
all (95,747)	240.76	7.3%	2.3°
50,000	259.86	7.9%	5°
25,000	298.80	9.1%	7.3°
6000	432.06	13.2%	12.7°

**Table 5:** Statistical Results of the Proposed Method.

Average Results Deviation of BS Location	Average Error of Azimuth Estimation	Accuracy of BS Type Estimation
11.5%	25.7	99.9%
